# Neural lesions in obstetrics: a diagnostic tree

**DOI:** 10.1186/s40981-022-00529-0

**Published:** 2022-06-07

**Authors:** Hector J. Lacassie, Patricio Mellado, Juan Pablo Cruz

**Affiliations:** 1 División de Anestesiología, Facultad de Medicina, Pontificia Universidad Católica de Chile, Marcoleta 377, 4th floor, Santiago, Chile; 2 División de Neurociencias, Facultad de Medicina, Pontificia Universidad Católica de Chile, Marcoleta 367, Santiago, Chile; 3 División de Imágenes, Laboratorios y Patologías, Facultad de Medicina, Pontificia Universidad Católica de Chile, Marcoleta 377, 5th floor, Santiago, Chile

To the Editor:

We read with great interest the case report by S. Shimizu, describing a unilateral radiculopathy due to adhesive arachnoiditis after spinal anesthesia for emergency cesarean section. His analysis concludes that there was a localized arachnoiditis of the left L5-S1 nerve roots, based on clinical findings and MRI imaging [1].

We were puzzled by the clinical presentation and evolution of symptoms. It began with hypoesthesia of the lateral aspect of the left thigh, resembling meralgia paresthetica, involving the femoral lateral cutaneous nerve (L2-3-4 nerve roots). They also noted motor weakness on dorsiflexion and plantar flexion of the left foot, suggesting lumbosacral plexus involvement (L4-S4). There is no other clinical description to allow a definite diagnosis. Also, MRI findings are equivocal and may not support the diagnosis of arachnoiditis since nerve roots are not adherent and the dural thickening is most likely due to a chemical shift artifact (type 1).

We believe this is a case report of foot drop syndrome in a patient that may have hereditary neuropathy with liability to pressure palsy that can be better described by ruling out positive and negative findings. This clinical dilemma is not unusual, especially in obstetrical patients where neural palsies are due to pregnancy or from obstetrical causes, and seldom from anesthesia origin [2]. We present a scheme that can help discriminate clinically different diagnoses.

Clinical assessment for post cesarean section foot drop syndrome.

1. Anamnesis
  - Previous spine disease (disk herniation, hereditary neuropathy with liability to pressure palsy, diabetes, etc.)
  - Labor:
    i. Leg support malposition: common fibular nerve palsy
    ii. Prolonged labor stages and forced leg positions: femoral nerve or lateral cutaneous femoral nerve palsy (meralgia paresthetica)
    iii. Instrumental delivery: sacral plexus palsy
  - Cesarean section:
    i. Surgical retractors: nerves or sacral plexus palsies
    ii. Leg malposition: femoral nerve or lateral cutaneous femoral nerve palsy (meralgia paresthetica)
  - Neuraxial anesthesia: Cauda equina syndrome, conus medullaris syndrome, or spinal lesion due to direct puncture, pharmacological damage, or infection
2. Neurological assessment
  - Motor function
    i. No flexion or eversion of the leg + hypoesthesia of leg and foot: common fibular nerve
    ii. No flexion or eversion of the leg + no abduction of the thigh (gluteus medius muscle): L5 nerve root palsy
    iii. No flexion or eversion of the leg + sphincter incontinence: cauda equina syndrome or lumbosacral palsy
  - Sensory function
    i. Leg and foot hypoesthesia only: superficial fibular nerve
    ii. Leg and foot hypoesthesia + abolished calcaneal (Achiles’) tendon reflex: sciatic nerve palsy or lumbosacral plexus palsy
    iii. Bilateral leg hypoesthesia: Cauda equina syndrome or conus medullaris syndrome

## Data Availability

Not applicable.
